# Aging and the Environment: A Research Framework

**DOI:** 10.1289/ehp.7569

**Published:** 2005-05-26

**Authors:** Andrew M. Geller, Harold Zenick

**Affiliations:** National Health and Environmental Effects Laboratory, Office of Research and Development, U.S. Environmental Protection Agency, Research Triangle Park, North Carolina, USA

**Keywords:** aging, environmental health, exposure, frailty, microenvironment, older adults, pharmacodynamics, pharmacokinetics, polypharmacy

## Abstract

The rapid growth in the number of older Americans has many implications for public health, including the need to better understand the risks posed to older adults by environmental exposures. Biologic capacity declines with normal aging; this may be exacerbated in individuals with pre-existing health conditions. This decline can result in compromised pharmacokinetic and pharmacodynamic responses to environmental exposures encountered in daily activities. In recognition of this issue, the U.S. Environmental Protection Agency (EPA) is developing a research agenda on the environment and older adults. The U.S. EPA proposes to apply an environmental public health paradigm to better understand the relationships between external pollution sources → human exposures → internal dose → early biologic effect → adverse health effects for older adults. The initial challenge will be using information about aging-related changes in exposure, pharmacokinetic, and pharmacodynamic factors to identify susceptible subgroups within the diverse population of older adults. These changes may interact with specific diseases of aging or medications used to treat these conditions. Constructs such as “frailty” may help to capture some of the diversity in the older adult population. Data are needed regarding *a*) behavior/activity patterns and exposure to the pollutants in the microenvironments of older adults; *b*) changes in absorption, distribution, metabolism, and excretion with aging; *c*) alterations in reserve capacity that alter the body’s ability to compensate for the effects of environmental exposures; and *d*) strategies for effective communication of risk and risk reduction methods to older individuals and communities. This article summarizes the U.S. EPA’s development of a framework to address and prioritize the exposure, health effects, and risk communications concerns for the U.S. EPA’s evolving research program on older adults as a susceptible subpopulation.

Demographics of the United States are changing rapidly. By 2030, the number of individuals older than 65 will more than double to 71.5 million [U.S. Administration on Aging (U.S. AoA) 2004]; one of every five Americans will be older than 65 [U.S. Department of Commerce (U.S. DoC) 1996]. This growth in the number of older Americans has major implications from both human and ecologic health perspectives. For human health, there will be an increasing need to understand the impact that environmental exposures and conditions might have on individuals as they enter later stages of life. Equally important will be the need to understand the implications for, or impact on, ecologic resources associated with accommodating the residential, medical, recreational, and transportation needs of this population. This document presents a preliminary framework for research to assist the U.S. Environmental Protection Agency (EPA) to better *a*) delineate the special susceptibilities associated with the aged compared with the healthy younger adult population, *b*) identify gaps in knowledge, and *c*) provide a starting point to establish research priorities. Given that the impact of the transition of “baby boomers” into senior citizens will rapidly accelerate in the next 5–10 years, opportunities exist to conduct research in the interim that will help to better inform decisions made by policy makers, institutions, and individual citizens. For the U.S. EPA, this work will provide a scientific rationale for decisions on how to appropriately incorporate the differential sensitivity of aging adults into environmental risk assessment, decisions, and actions. A parallel framework for considering the ecologic and resource use implications of the growing population of older adults is also being developed (U.S. EPA 2005b).

This article is a brief review based on primary and secondary sources that address the susceptibility of older adults to effects of environmental exposures and describes a wide array of research priorities. Two U.S. EPA-sponsored activities have also greatly informed this document. The first was the workshop “Differential Susceptibility and Exposure of Older Persons to Environmental Hazards’’ convened by the National Academy of Sciences (NAS) in December 2002 (NAS 2003). In addition, the U.S. EPA invited public comments on environmental hazards that may affect the health of older adults in states and local communities at six public listening sessions held throughout the United States in the spring of 2003 (U.S. EPA 2003a) and from comments sent directly to the U.S. EPA (2005a). The priorities that emerged from the NAS workshop and the public listening sessions were similar to those previously described by the NAS and the International Programme on Chemical Safety (IPCS) (Baker and Rogul 1987; IPCS 1993; National Research Council 1987).

## Environmental Public Health Framework

A research program focused on the aging population is consistent with the priority that the U.S. EPA gives to susceptible subpopulations in its risk assessment/risk management processes. Several of the U.S. EPA’s statutes mandate such considerations [e.g., Clean Air Act of 1970 (1970), Food Quality Protection Act of 1996 (1996), Safe Drinking Water Act of 1974 (1974)]. As with the Agency’s well-established programs to assess risk to children (U.S. EPA 2000), a program focused on the aging population must consider factors that affect susceptibility associated with various life stages. For example, parallel assumptions are that individuals may be at greater risk at certain life stages as a result of modified pharmacokinetic and pharmacodynamic capacity and different exposure conditions. Susceptibility of older adults can be defined by qualitative or quantitative differences. Qualitative differences mean that exposure-related adverse health outcomes are present in older adults that are not present in younger individuals. Quantitative differences mean that a toxicologic outcome may be observed at lower doses, have a greater severity, or have a shorter latency to onset in older individuals than in young adults.

It is important to recognize that variability is a hallmark of the aging population (National Research Council 1987; Schmucker 1998, 2001; Vestal 1989), so it is likely that members of an aging population will exhibit variability in their responses to environmental agents. Thus, it follows that research will not generate a “one-size-fits-all” set of recommendations for risk management/health prevention actions. For example, at least three subpopulations can be identified: *a*) healthy individuals with normal but possibly diminishing capacities; *b*) individuals confronted with the emergence of disease/illness associated with later years (e.g., Alzheimer disease, age-related sensory losses); and *c*) individuals already afflicted with disease/illness entering this period of life (e.g., cardiovascular disease, respiratory disease, thyroid deficiency, diabetes).

One construct that may account for some of the variability of the aging population is that of the “fit” versus “frail” elderly (Crome 2003; O’Mahoney 2000). Fit refers to older individuals who are able to independently perform the daily activities in the community; frailty refers to older adults who may not be independently mobile and may be dependent on others to carry out daily activities, and are often in institutionalized care. The roots of frailty may lie in alterations to multiple physiologic systems (Walston 2004). It connotes diminished reserve capacity, diminished resistance to stressors, and increased health risk (Bortz 2002; Fried et al. 2001; Schuurmans et al. 2004). This construct may work to summarize the overall effects of the many conditions that affect health in the elderly because frailty has been shown to reduce both pharmacokinetic and pharmacodynamic functions (Kinirons and O’Mahoney 2004; Walston et al. 2002). It may also help to identify individuals or subgroups among the heterogeneous older adult population that might be susceptible to environmental agents because it has been shown to be a better predictor of adverse outcomes in older adults than chronologic age (Schuurmans et al. 2004).

**Figure 1:**
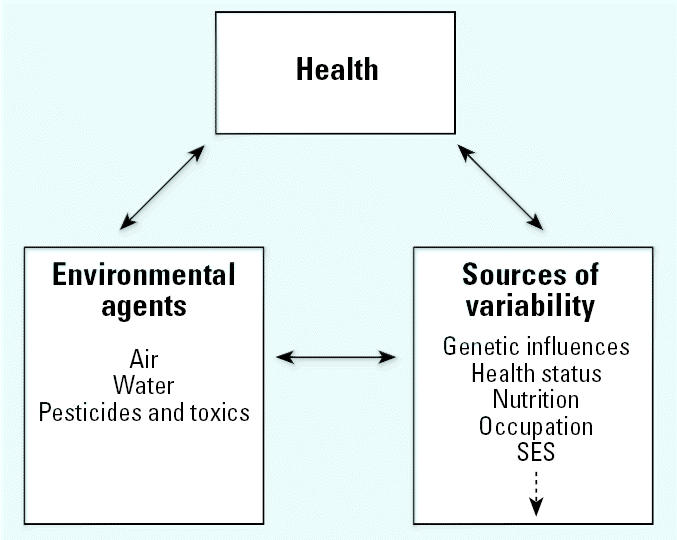
The interactions of environmental health, exposure, and additional sources of variability with aging broadly define the proposed dimensions for research on the health effects of exposure to environmental agents in older adults. SES, socioeconomic status. The dashed arrow signifies that many more items could be included along with the sources of variability listed.

Additional factors, including sex, socioeconomic status, cultural differences, lifestyle, nutrition, exposure history, and geographiclocation, may also be used to stratify or characterize the population of older adults. These sources of variability can be considered cross-cutting influences because they may be important determinants of the exposures experienced as well as the health outcomes. For example, these factors may modify the environmental exposure experienced by older individuals through effects on behavior and lifestyle choices, by influencing residential choices, daily habits, and activity patterns. They can also affect how the body responds to potentially threatening environmental exposures, influencing both what the body does to environmental toxicants (pharmacokinetics) and what those toxicants do to the body (pharmacodynamics) (Figure 1).

These cross-cutting factors are potential mediators of susceptibility, some of which are more the province of other federal agencies. One goal of this research framework is that it will be a basis for fostering collaborative research with these sister agencies.

**Figure 2:**
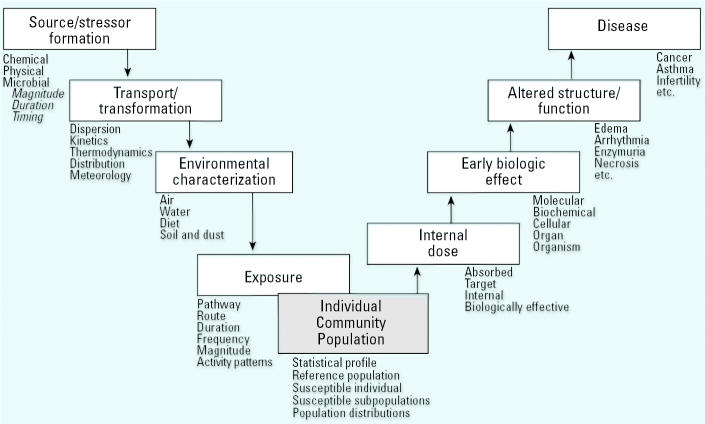
Environmental public health continuum used by the U.S. EPA for strategic planning of research. Modified from the U.S. EPA (2003c).

An environmental public health continuum (Figure 2) that has been used previously by the U.S. EPA (2003b, 2003c) to aid in the development of broad research strategies has also been used in this article. Along this continuum are the cascade of events beginning with the source through adverse health effects. Research is directed to helping researchers understand the determinants influencing each component along the continuum and, equally important, the predictive linkages between components. The continuum is also of heuristic value in arraying ongoing research and identifying major gaps to help set priorities. The following sections concentrate on the contributions of the components identified as external exposure, internal dosimetry, and health outcomes (early biologic effects and the manifestation of disease) to the potential susceptibility of older adults. These data, in turn, provide a basis for risk management and health promotion.

### Exposure

Exposure is the contact between an environmental agent and a target. In exposure assessments, exposure is usually quantified as the product of the concentration of the agent in environmental media with which an individual comes in contact (e.g., air, water, food) and the time the individual is in contact with the environmental agent. The behaviors that bring an individual into contact with an environmental agent are important determinants of the level of exposure. For example, inhalation exposures depend on the microenvironments where people spend their time. The term “microenvironment” refers to the immediate surroundings of an individual that can be treated as homogeneous or well characterized in the concentrations of an agent (e.g., home, office, automobile, kitchen, store). Understanding microenvironments is critical because the highest personal exposures may occur where little time is spent but contaminant levels are high. For example, up to 35% of an individual’s daily exposure to particulate matter (PM) may come from microenvironments where only 4–13% of time is spent (Rea et al. 2001). Recent data have been published on the personal exposures of older adults to PM (Lippman et al. 2003; Rodes et al. 2001; Williams and Wallace 2002).

The sources and pathways of exposure as well as exposure locations may differ in older adults compared with younger adults. Current characterizations of the older adult population suggest that older adults spend more time indoors than younger adults, particularly in residences, and show marked similarities to the very young (0–4 years of age) in where they spend their time and in their types of environmental exposures (Jenkins et al. 1992; Klepeis et al. 1996; Williams et al. 2000a, 2000b). Time spent indoors is important because many hazardous air pollutants occur at higher concentrations indoors, thus potentially exacerbating exposure to indoor air pollutants (Kinney et al. 2002; Saarela et al. 2003; Spengler et al. 1985; U.S. EPA 1998). There are, however, differences within the older population, again demonstrating that this is a heterogeneous group. For example, older adults in similar residential situations in different locations (Baltimore, Maryland vs. Fresno, California) spend different amounts of time indoors (Rea et al. 2001). Differences in activity patterns such as cooking, which may have implications for PM exposure, can also be seen between older adults in residential retirement centers (Williams et al. 2000b) and the broader population (Klepeis et al. 1996).

### Dose

The goal of research on internal dosimetry is to understand the effects of physiologic and biochemical changes with age on target tissue dose for a given exposure. This work focuses on the pharmacokinetic processes of absorption, distribution, metabolism, and elimination (ADME) that determine the dose of an environmental agent that reaches a target organ. Many age-related differences in drug and toxicant responsiveness appear to be based on altered ADME (Table 1) (Birnbaum 1991; Clewell et al. 2002, 2004; Mayersohn 1994; O’Mahoney 2000; von Moltke et al. 1995). Changes in these processes mean that the same external dose may result in a very different internal dose or distribution to different target organs in older adults.

**Figure 3:**
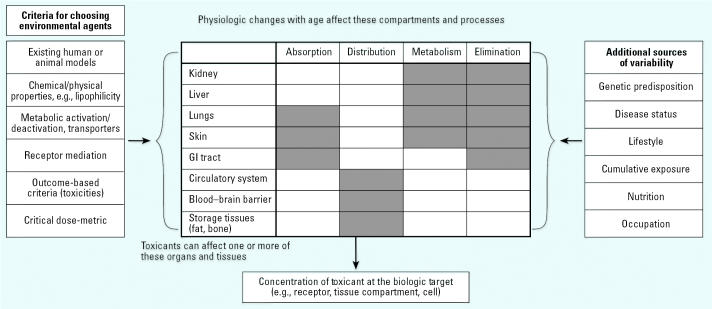
Predictive modeling to identify the pharmacokinetic parameters that most affect outcomes such as tissue dosimetry and toxicity will consider prototype toxicants chosen according to a set of criteria (left), physiologic compartments and processes (center), and additional sources of variability that affect physiologic function (right). GI, gastrointestinal. All of these contribute to the determination of the level of a toxicant at its biologic target. Shaded boxes indicate which of the body’s compartments are mainly involved in ADME of environmental exposures, recognizing that almost all tissues have some metabolic capacity.

The current pharmaceutical literature indicates that fit older adults are quite similar to fit younger adults in pharmacokinetic parameters, with the general exceptions of decreased renal excretion and hepatic processing, secondary to changes in hepatic blood flow and liver volume (O’Mahoney 2000). It is notable, however, that pharmacokinetic function is decreased in frail older individuals. Disease, physical trauma, and changes in nutritional status (O’Mahoney 2000; Walston 2004) can alter many pharmacokinetic factors (Figure 3).

#### Absorption.

Absorption occurs principally via the gastrointestinal tract, the respiratory tract, or the skin. There are no marked age-related changes in gut absorption after oral exposure. One exception involves decreased acid production in the stomach, which reduces the dissolution of basic compounds (Mayersohn 1994; O’Mahoney 2000; Schmucker 1985). The inhalation pathway may show changes in absorption or deposition due to age- or disease-related changes in lung volume, ventilation rate, and alveolar elasticity (Clewell et al. 2002; Lippman et al. 2003). For example, changes due to airway obstruction that accompany chronic obstructive pulmonary disease result in deeper penetration of PM and a higher rate of particle deposition (Brown et al. 2002; Kim and Kang 1997). Changes in dermal structure and function with aging may alter dermal absorption such that the ability of the skin to exclude certain compounds may be reduced with aging. This reduction in barrier function is most likely to accompany pre-existing conditions that place the skin under stress—the skin of older adults recovers from stresses significantly more slowly compared with that of younger adults (Elias and Ghadially 2002; Ghadially et al. 1995; Ye et al. 2002).

#### Distribution.

Distribution of a chemical throughout the body can be affected by many factors, including body composition, blood flow, and plasma binding proteins (Clewell et al. 2002). Changes in body composition (Table 1) can result in reduced volume of distribution or increased half-lives for xenobiotic compounds, depending on whether compounds are soluble in lipids or water (O’Mahoney 2000; Schmucker 1985; von Moltke et al. 1995). Changes in plasma protein binding may also be critical (Table 1) because the main factor determining the effect of a compound in the body is the free, unbound fraction of that compound (Birnbaum 1991; O’Mahoney 2000; von Moltke et al. 1995). Age-related reductions in serum albumin can increase the serum-free fraction of lipophilic compounds, whereas age-or disease-related increases in α_1_-acid glycoprotein affect the binding of basic compounds (Clewell et al. 2002).

Another potential area of concern is changes in the blood–brain barrier with aging, resulting in increased permeability of the cerebral microvasculature to toxicants that could result in neurodegenerative disease. Most data currently indicate that there are no significant changes in permeability with normal aging (Shah and Mooradian 1997). Diseases often associated with aging, however, such as diabetes, hypertension, and cerebral ischemic events, may compromise this barrier function (Johansson 1998; Mooradian 1997; Starr et al. 2003; Wisniewski and Lossinsky 2002). This may be important in understanding the environmental etiology of conditions such as parkinsonism, which has been linked to exposure to some compounds that ordinarily show limited penetration of the blood–brain barrier (Brooks et al. 1999; Thiruchelvam et al. 2003).

#### Metabolism.

The liver is the major metabolic organ in the body, and studies show that levels of liver activity drop with aging (Youssef and Badr 1999). This decreased activity could result in slowed detoxification of some compounds and reduced excretion rates, which can result in higher effective circulating levels and longer half-lives.

Although it was initially thought that the age-related decrease in metabolism was due to changes in the activity of liver enzymes, current data indicate that most age-related changes in hepatic activity are due to declines in liver volume and blood flow with age (O’Mahoney 2000; Schmucker 2001; Vestal 1989). There are few known significant changes in the levels of activity of metabolic enzymes with normal aging (Schmucker 1998, 2001; Shimada et al. 1994), but many gaps still remain in the understanding of aging-related metabolic changes (Clewell et al. 2002). The disposition of xenobiotics is also affected by transporters such as P-glycoprotein (Pgp) and multidrug-resistance–associated protein. There is increased Pgp expression in lymphocytes of older adults; it has been suggested that this may have an effect on metabolism and drug interactions (Gupta 1995; Kinirons and O’Mahoney 2004; McLean and LeCouteur 2004). The effect of aging on Pgp function throughout the body is still unknown.

The role of the liver enzymes is critical to another aspect of the issue of age-related changes in metabolism: polypharmacy, the administration of two or more pharmaceutical compounds to an individual. Studies show that 90% of people older than 65 take one or more medications daily, with most taking two or more, and residents of nursing homes or care facilities average six to eight medications per individual (Prybys et al. 2002; Vestal 1997). Because the same biologic processes “clear” medications and environmental toxicants, there is concern that older adults who take multiple medications may be at increased risk of adverse reactions between medications and concurrent or subsequent environmental exposures. Either induction or inhibition of metabolic enzymes by environmental chemicals (Borlakoglu and Haegele 1991; Butler and Murray 1997; Thummel and Wilkinson 1998; Youssef and Badr 1999) could alter the body’s critical processing of pharmacologic agents (Shimada et al. 1994; Thummel and Wilkinson 1998). Conversely, metabolic processes can make some environmental chemicals more biologically active, as in the case of some carcinogens or pesticides (Buratti et al. 2003; Guengerich and Shimada 1991; Sams et al. 2000). Therefore, exposure to these compounds, in conjunction with medications that may induce higher levels of enzyme activity, could result in greater toxicity (U.S. Food and Drug Administration 2002). Polypharmacy may also affect plasma protein binding if competitive displacements occur (Herrlinger and Klotz 2001; Mayersohn 1994; Rolan 1994).

#### Elimination.

The elimination of toxicants and their metabolites is affected by age-related changes in hepatic function, described above, and by decreased kidney function. A decrease in the rate of renal clearance results in an increase in the elimination half-life of a compound. Renal changes observed with age include a decrease in mass of the kidneys, a reduction in the size and number of nephrons, a decrease in renal blood flow, and reductions in glomerular filtration rate, renal plasma flow, and tubular function (Mayersohn 1994; O’Mahoney 2000). In addition, the alterations in pulmonary function that affect absorption of gases and volatile compounds also will affect their excretion through the pulmonary route (Birnbaum 1991). There is also evidence that bile flow and biliary transport is reduced with aging, thus reducing excretion through that route (Birnbaum 1991).

### Health Outcomes

There are clear examples of increased health risk associated with environmental exposures of older individuals. Research on PM has shown significant associations between cardiopulmonary morbidity and pollutant levels (Bateson and Schwartz 2004; Zanobetti et al. 2000). Older adults are also more vulnerable to gastrointestinal disease from waterborne pathogens (Naumova et al. 2003). However, aging-related changes in pharmacodynamic processes that may limit the body’s ability to maintain homeostasis and respond to injury have been studied less extensively than the pharmacokinetic changes (Vestal 1997). Older adults may be more susceptible to toxicants in the environment because they have a decreased ability to compensate for the effects of environmental insult, that is, a reduced “reserve capacity.” The mechanisms underlying a decreased compensatory ability may be similar across organ systems but may be expressed differently in those organ systems. For example, the same processes that play a major role in cancer initiation and promotion may also play an important role in cognitive decline with aging (Lu et al. 2004). These processes include DNA damage in promotor regions of genes with reduced expression and a reduction in base-excision DNA repair associated with oxidative stress and impaired mitochondrial function. Additional pharmacodynamic changes include age-related changes in receptor numbers, sensitivity, and up- and down-regulation, as well as altered signal transduction, reduced numbers of neurons, and changes in calcium homeostasis (Goldman et al. 1994; Michalek et al. 1990). Alterations to other mechanisms of plasticity or homeostasis include reduced immune response, altered response to oxidative stress (Kodavanti 1999), and reduced DNA repair and anti-proliferation mechanisms (Frohman 1994; Lu et al. 2004; Rehman and Masson 2001). Aging also results in changes in neuroendocrine and neurotransmitter levels (dopamine, GABA, glutamate) along with alterations in the thyroid–pituitary axis; decreases in the production of sex steroids, growth hormone, and insulin-like growth factor; and increases in glucocorticoids and cytokines (Frohman 1994; Rehman and Masson 2001 Smith et al. 2004). These age-related alterations in function may then contribute to the increased vulnerability of older individuals to a variety of environmentally linked adverse health outcomes. Examples include neurotoxicity (Kodavanti 1999; Spencer 1990; Weiss 1990), cancer (Akman 2003), cardiovascular and pulmonary morbidity (Lippman et al. 2003; Reaven 2003), inflammatory responses (McCord 2003), and gastrointestinal effects associated with increased susceptibility to waterborne pathogens (Naumova et al. 2003).

## Research Recommendations

One of the challenges in conducting research on aging is addressing the great diversity of health and exposure conditions of the older adult population. To be responsive to public health needs, it is important to be able to predict which older adults, defined not only by age but also by the influence of the cross-cutting factors discussed above, will be most susceptible and to which environmental agents. Employing the environmental public health continuum (Figure 2), the following are research areas under initial consideration by U.S. EPA.

### *Exposure: provide data for use in source-to-dose exposure models applicable to older adult populations and information specific to older adults for the U.S. EPA* Exposure Factors Handbook *(U.S. EPA 1997).*

Initial steps include the identification of susceptible subgroups of older adults on the basis of exposure and activity modeling. Exposure models will be derived from information on chemical and biologic stressors, geographic location, and health status drawn from existing databases at the U.S. EPA. Other resources to be “mined” include age-specific census, occupational, dietary, and product safety data. Research on activity patterns and microenvironments of importance to the elder population includes characterization of time spent indoors, recreational choices, occupation, control over the environment in group housing, and the effects of reduced mobility, lifestyle choices, and isolation. This initial modeling and compilation of data will help researchers identify and prioritize data gaps. This will, in turn, generate hypotheses and guide further development of the database and refinement of models for assessing the degree to which susceptibility in older adults is due to differences between older and younger adults in activity and in exposures to harmful environmental agents.

### Dose: determine the contribution of age-related alterations in pharmacokinetics to the susceptibility of older adults.

The initial steps in this research will also be model-driven in that sensitivity analysis will help to determine which factors, such as changes in particular metabolic enzymes, transport processes, or excretion functions, are most important for particular classes of chemicals. Modeling should also define the magnitude of change necessary for a factor to alter the exposure–dose–response relationship for prototype chemicals. This will help to narrow the focus for empirical research on physiologic and biochemical parameters that will have the greatest effect in older adults by overlaying those factors on the ones that are known to change with aging and with diseases of aging. It may also help to define which diseases or medications might be expected to increase susceptibility to effects of environmental exposures (Figure 3). Susceptibility due to pharmaceutical use will be informed by our understanding of the common mechanisms underlying the metabolism of pharmaceutical compounds and environmental agents. In addition, identification of critical kinetic factors will aid in the evaluation and development of animal or *in vitro* models because effects noted in rodent models of aging have not always accurately reflected changes present in aging humans (e.g., Schmucker 2001).

### Health outcomes: determine the relationship between exposure to environmental agents and adverse health effects in aging populations.

Data from mechanistic research conducted to understand the age-dependence of pharmacodynamic processes such as protective, repair, compensatory, and plasticity mechanisms across organ systems can be applied to the question of whether mode of action information can predict which subpopulations are susceptible to the effects of environmental agents. As with the pharmacokinetic approach, this work will identify and prioritize the processes or mechanisms that confer susceptibility on aging adults and match these with environmental agents presumed to operate through similar putative mechanisms.

### Risk communication: develop a strategy for communication of risk, risk management, and public health intervention.

This will likely include the dissemination of information to and through environmental and health professionals, state and local governments, developers of senior communities, and the broad community of professionals, organizations, and associations involved with aging issues. Effective communication must consider the social and cognitive strategies most appropriate for older adults (Helmuth 2003; Park 2002).

## Conclusion

The research framework we describe in this article focuses on the potential for interactions between aging and environmental exposures to produce adverse health effects in older adults. This research program will generate data on exposures that the aging population experiences and the subsequent pharmacokinetic and target organ responses, with the goal of providing a better understanding of the environmental health risks associated with aging in healthy or compromised older adults. These data will be used to generate models and guidance on how to appropriately incorporate the differential susceptibility of this heterogeneous subpopulation into health promotion and intervention strategies to ameliorate risk from environmental exposures.

Now, still a few years away from the cresting of this demographic wave, is the time to anticipate, accommodate, and manage the environmental risks associated with this inevitable shift in American demographics toward an aging society.

## Figures and Tables

**Table 1:** Pharmacokinetic changes that may contribute to increased susceptibility in older persons.

Process	Pharmacokinetic changes in aging adults
Absorption	No significant changes in gastric absorption; decline in gastric acid production
	Changes in dermal absorption, barrier function
	Changes in lung volume, elasticity, ventilation rate
Distribution	Change in body composition
	Decreased total body water in older adults results in decreased volume of distribution/higher serum levels for polar compounds
	Decreased muscle mass and increased relative adipose levels result in higher accumulation of lipophilic compounds and slower clearance
	Plasma protein binding—decrease in plasma albumin (which bind acidic compounds), increase in α_1_-glycoprotein (bind basic compounds)
	Potential for increased permeability of blood–brain barrier with concurrent disease (diabetes, hypertension, cerebrovascular ischemia)
Metabolism	Reduced liver volume and liver blood flow
	Minor effects on phase I and II metabolism in healthy aging
	Significant metabolic effects in conjunction with frailty/age-associated disease
	Decline in specific cytochrome P450 content
	Polypharmacy—interactions of environmental toxicants with therapeutic compounds, herbal supplements, and diet due to shared metabolic pathways, and/or induction or inhibition of metabolic enzymes and/or transporters
Excretion	Reduced renal function
	Reduced blood flow
	Reduced glomerular filtration
	Reduced renal MFO activity, inducibility
	Reduced biliary excretion
	Reduced pulmonary excretion

MFO, mixed-function oxidase.
